# Evidence for pre‐symptomatic transmission of coronavirus disease 2019 (COVID‐19) in China

**DOI:** 10.1111/irv.12787

**Published:** 2020-08-07

**Authors:** Xiang Ren, Yu Li, Xiaokun Yang, Zhili Li, Jinzhao Cui, Aiqin Zhu, Hongting Zhao, Jianxing Yu, Taoran Nie, Minrui Ren, Shuaibing Dong, Ying Cheng, Qiulan Chen, Zhaorui Chang, Junling Sun, Liping Wang, Luzhao Feng, George F. Gao, Zijian Feng, Zhongjie Li

**Affiliations:** ^1^ The Chinese Center for Disease Control and Prevention Beijing China

**Keywords:** China, COVID‐19, epidemiology, pre‐symptomatic Transmission

## Abstract

**Background:**

Between mid‐January and early February, provinces of mainland China outside the epicentre in Hubei province were on high alert for importations and transmission of COVID‐19. Many properties of COVID‐19 infection and transmission were still not yet established.

**Methods:**

We collated and analysed data on 449 of the earliest COVID‐19 cases detected outside Hubei province to make inferences about transmission dynamics and severity of infection. We analysed 64 clusters to make inferences on serial interval and potential role of pre‐symptomatic transmission.

**Results:**

We estimated an epidemic doubling time of 5.3 days (95% confidence interval (CI): 4.3, 6.7) and a median incubation period of 4.6 days (95% CI: 4.0, 5.2). We estimated a serial interval distribution with mean 5.7 days (95% CI: 4.7, 6.8) and standard deviation 3.5 days, and effective reproductive number was 1.98 (95% CI: 1.68, 2.35). We estimated that 32/80 (40%) of transmission events were likely to have occurred prior to symptoms onset in primary cases. Secondary cases in clusters had less severe illness on average than cluster primary cases.

**Conclusions:**

The majority of transmissions are occurring around illness onset in an infected person, and pre‐symptomatic transmission does play a role. Detection of milder infections among the secondary cases may be more reflective of true disease severity.

## 1 INTRODUCTION

A novel coronavirus named “severe acute respiratory syndrome coronavirus 2” (SARS‐CoV‐2) was first identified in January 2020 as the pathogen responsible for a cluster of cases of atypical pneumonia in Wuhan, a large city located in Hubei province in central China.^1^, ^2^ Genetic analysis of the virus indicates that it originated from a bat coronavirus.^2^ SARS‐CoV‐2 is considered distinct from SARS‐CoV or MERS‐CoV, and coronavirus disease 2019 (COVID‐19) caused by it has rapidly become a global health concern.^3^ Incidence of infections slowly increased through January 2020 with a reproductive number estimated to be in the range 2.2‐3.6.^4^, ^5^, ^6^, ^7^ Starting in mid‐January 2020, COVID‐19 cases began to be identified in other cities in China and also in other countries.^8^, ^9^ A number of studies suggested the probable phenomenon of COVID‐19 transmissions during incubation period,^10^, ^11^, ^12^ these studies used cluster cases in limited number of families for analysis, and the relative frequency of pre‐symptomatic transmission was not quantified. Moreover, there was an analysis on publicly available data indicating the existence of negative serial intervals, also implying pre‐symptomatic transmission.^13^ Here, we retrospectively analyse data on cases identified outside of Hubei province through the Chinese Public Health Event Surveillance System at the early stage of transmission in China, in order to provide insights on the transmission dynamics of COVID‐19.

## 2 METHODS

Data were extracted from the epidemiological reports of the Chinese Public Health Event Surveillance System, through which the first confirmed case or potential outbreaks leading to clusters of suspected cases for each county were required to be investigated and reported. The extracted variables included demographic data, possible exposure and travel history, and clinical data using a structured form. The events reported by 29 January 2020 were included for data extraction. For events by 23 January 2020, which was the date of “locking down” Wuhan city, all events (168 cases) in the system were extracted, and we used these data to construct an epidemic curve and estimate the incubation period distribution. Of the events (281 cases) reported between 24 January and 29 January 2020, we focused on those events which included probable human‐to‐human transmission or epidemiologically linked cases, so that we could capture a larger number of these clusters for analyses of serial intervals, transmission events and comparative severity between primary cases and secondary cases. All cases included in our analyses were laboratory‐confirmed cases, and the case definitions followed the Novel Coronavirus Pneumonia Prevention and Control Protocol published by the National Health Commission.^4^, ^14^

Data collection and analysis complied with the Novel Coronavirus Pneumonia Prevention and Control Protocol issued by the National Health Commission of the People's Republic of China. It is part of a continuing public health outbreak investigation thus exempt from institutional review board approval.

We drew the epidemic curve by illness onset for cases who reported that they had been in Wuhan in the 14 days of preceding onset, and a separate epidemic curve for the other cases. Cases with onset dates closed to the end of epidemic curve may not be reported due to delays in seeking medical attention and consequent delays in laboratory testing. To allow for onset‐to‐reporting delays, we used the nowcasting approach described by van de Kassteele et al^15^ to jointly estimate the augmented case number on the most recent dates and the onset‐to‐reporting distribution. Based on 1000 augmented epidemic curves, then we fitted exponential growth models to obtain estimates of the growth rate and doubling time. We examine the characteristics of confirmed cases and compared the demographics, onset symptoms and results of some clinical tests between confirmed sporadic and cluster primary cases and cluster secondary cases.

For cases in residents of Wuhan who travelled to other provinces, the delay between the most recent date of departure from Wuhan and the illness onset date was taken as a lower bound on the incubation period with upper bound 21 days. For cases in individuals who were not residents of Wuhan, the period of visiting Wuhan in the preceding 14 days was compared with the illness onset date to construct a minimum and maximum incubation period. This information was used to estimate the incubation period distribution with a lognormal parametric distribution, fitting by maximum likelihood accounting for interval‐censoring in the exact date of infection^16^ and accounting for exponential growth in the epidemic increasing the risk of infection over time during the exposure window by weighting each day in the exposure window by the estimated growth rate.

We also obtained information on secondary cases that had not been to Wuhan but had close contact with another case that had been to Wuhan and were presumed to be infected by that particular case. In these pairs of primary and secondary cases, we fitted a normal distribution to the serial intervals between illness onset dates, allowing for negative and zero serial intervals, and correcting for growth rates in the early stage of an epidemic. Specifically, the parameters of the fitted normal distribution were corrected to Normal (µ′,σ) where µ′ = µ + σ^2^ρ for the epidemic growth rate R estimated below (personal communication, Neil Ferguson). Because information was available on the periods during which each secondary case had been exposed to their presumptive infector, we were able to identify cases where exposure is likely to have occurred before or after illness onset in the primary case infector. When the exposure window overlapped the onset date, we used our fitted incubation period distribution to resample 1000 infection times at random, excluding any that fell outside the exposure window, and counted the proportion of resampled times that fell before symptom onset in the infector.

We estimate effective reproductive number R_e_ of COVID‐19 in Wuhan by integrating the serial interval estimate described earlier to estimate the effective reproductive number in Wuhan based on the formula R = exp (ρµ′ − (1/2)ρ^2^σ^2^),^17^ assuming that exponential growth in cases linked to travellers from Wuhan would reflect the rate of exponential growth of infections in the originating city and approximating the generation time distribution by the serial interval distribution. We used a bootstrap approach to conduct resampling in each augmented epidemic curve and serial interval distribution to account for the uncertainty in the estimation in each step. This was not necessarily an estimate of the basic reproductive number R_0_, since some behavioural changes and preventive measures occurred in Wuhan after the discovery of the outbreak linked to the Huanan Seafood Wholesale Market in early January.^4^

## 3 RESULTS

We collected detailed information on 168 confirmed cases, including 145 and 23 reporting that they had or had not, respectively, been in Wuhan in the past 14 days. The demographics of the 145 and 23 cases were very similar (data not shown). The median age was 48 (range 10‐81) and 91 (54%) were male. Only three (2%) of the cases who had visited Wuhan also reported visiting the Huanan Seafood Wholesale Market.

**Figure 1:**
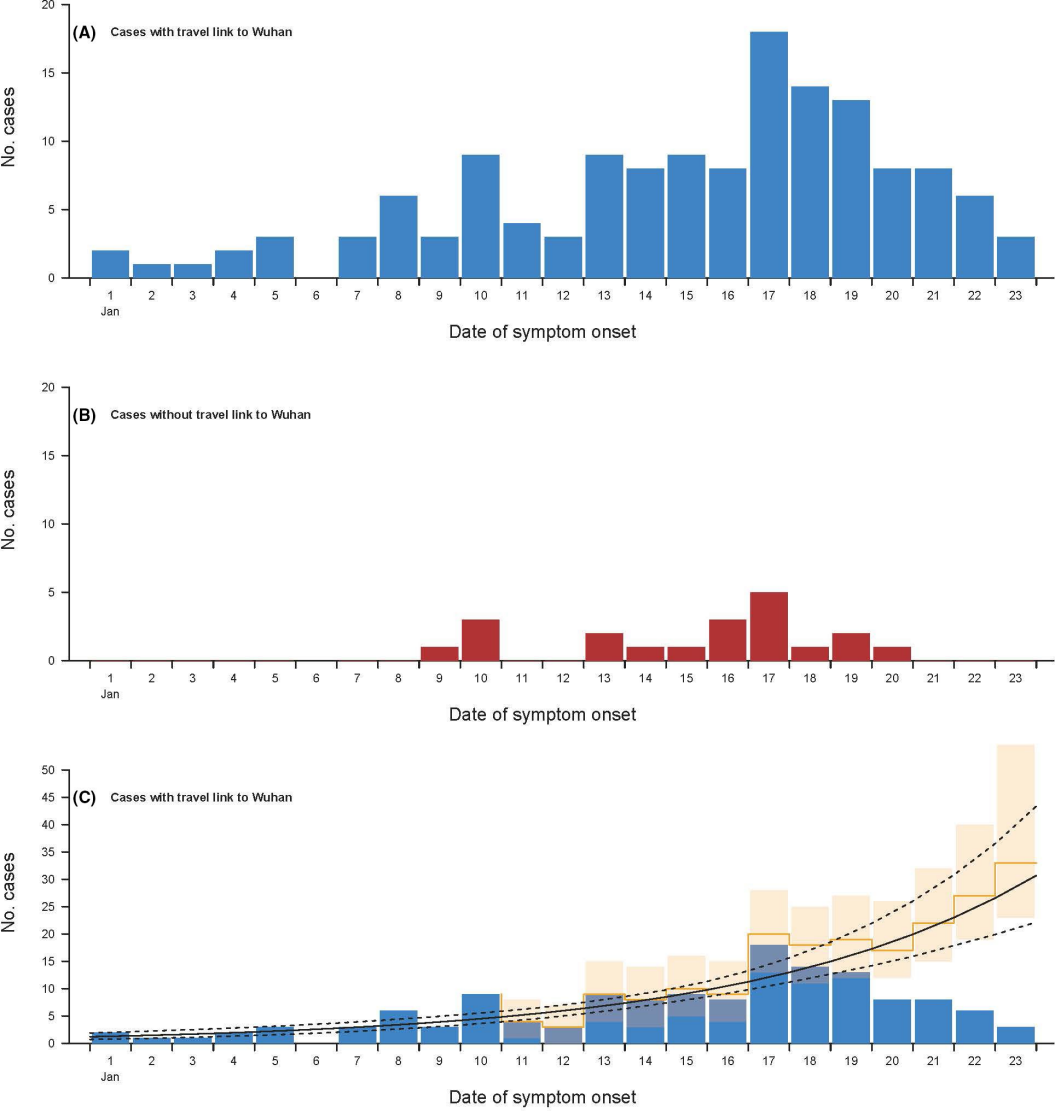
Panel A: occurrence by date of illness onset of cases identified outside of Hubei province in persons with a history of travel from Wuhan in the 14 d prior to onset. Panel B: occurrence by date of illness onset of cases identified outside of Hubei province in persons without a history of travel from Wuhan in the 14 d prior to onset. Panel C: augmented occurrence (yellow bars) by date of illness onset of cases identified outside of Hubei province in persons with a history of travel from Wuhan in the 14 d prior to onset with back filled cases considering delays between illness onset and seeking care and being tested

Figure 1 shows the epidemic curve by illness onset of the cases in persons that had been to Wuhan compared to cases in other persons that were presumed to represent onwards transmission. We used data augmentation to correct for reporting delays in cases with recent onset (Figure 1C) and estimated that the growth rate was 0.14 per day (95% CI: 0.11, 0.17), and the doubling time was 5.3 days (95% CI: 4.3, 6.7). In this analysis, the mean onset to reporting delay was estimated to be 5.7 days (95% CI: 5.3, 6.1).

**Figure 2:**
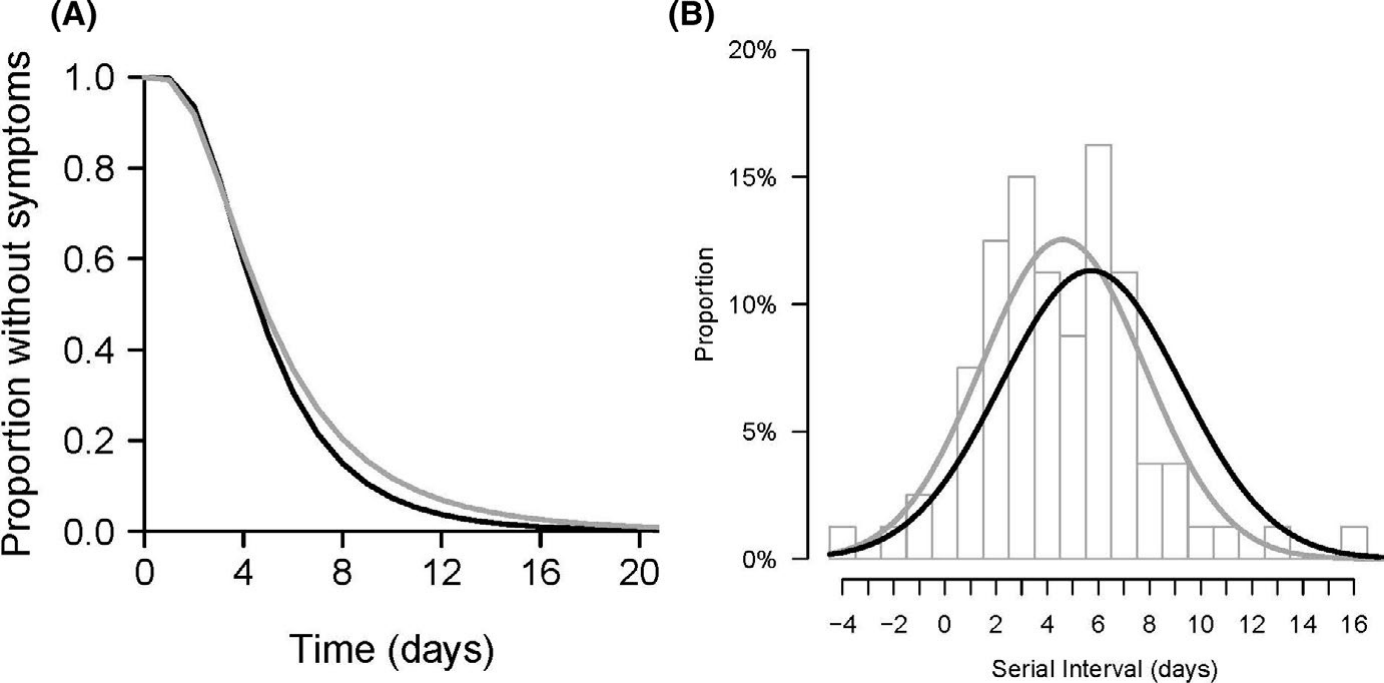
Panel A: incubation period distribution based on 98 cases identified outside Hubei province and reporting recent travel from Wuhan. The grey line indicates the empirical fitted lognormal distribution, and the solid black line indicates the fitted lognormal distribution after correction for epidemic growth. Panel B: serial interval distribution based on 80 observed transmission events. The grey bars show the raw data, the grey line indicates the empirical fitted gamma distribution, and the solid black line indicates the fitted gamma distribution after correction for epidemic growth

We obtained data on exposure windows for 98 cases in individuals that had been in Wuhan, by assuming that they had been infected while they were in Wuhan. We fitted a lognormal distribution to the data on exposure periods and onset dates, correcting for epidemic growth, and estimated that the incubation period had mean 5.3 days (95% CI: 4.6, 6.0), median 4.6 days (95% CI: 4.0, 5.2), 95th percentile 11.1 days (95% CI: 9.3, 12.8) and 99th percentile 16.1 days (95% CI: 12.9, 19.2) (Figure 2A).

We identified 64 clusters of infection among 21 provinces, in which person‐to‐person transmission from the primary case was thought to be the only explanation for the infection in the secondary case (ie no common exposure and no exposure to other infected persons). In total, there were 80 transmission events determined among which 64 pairs (80%) were family members who lived together, 11 (14%) occurred in those who had a meal together, 2 (3%) were colleagues working together, 2 (3%) were those who took the same vehicle, and 1 (1%) occurred among neighbours.

**Figure 3:**
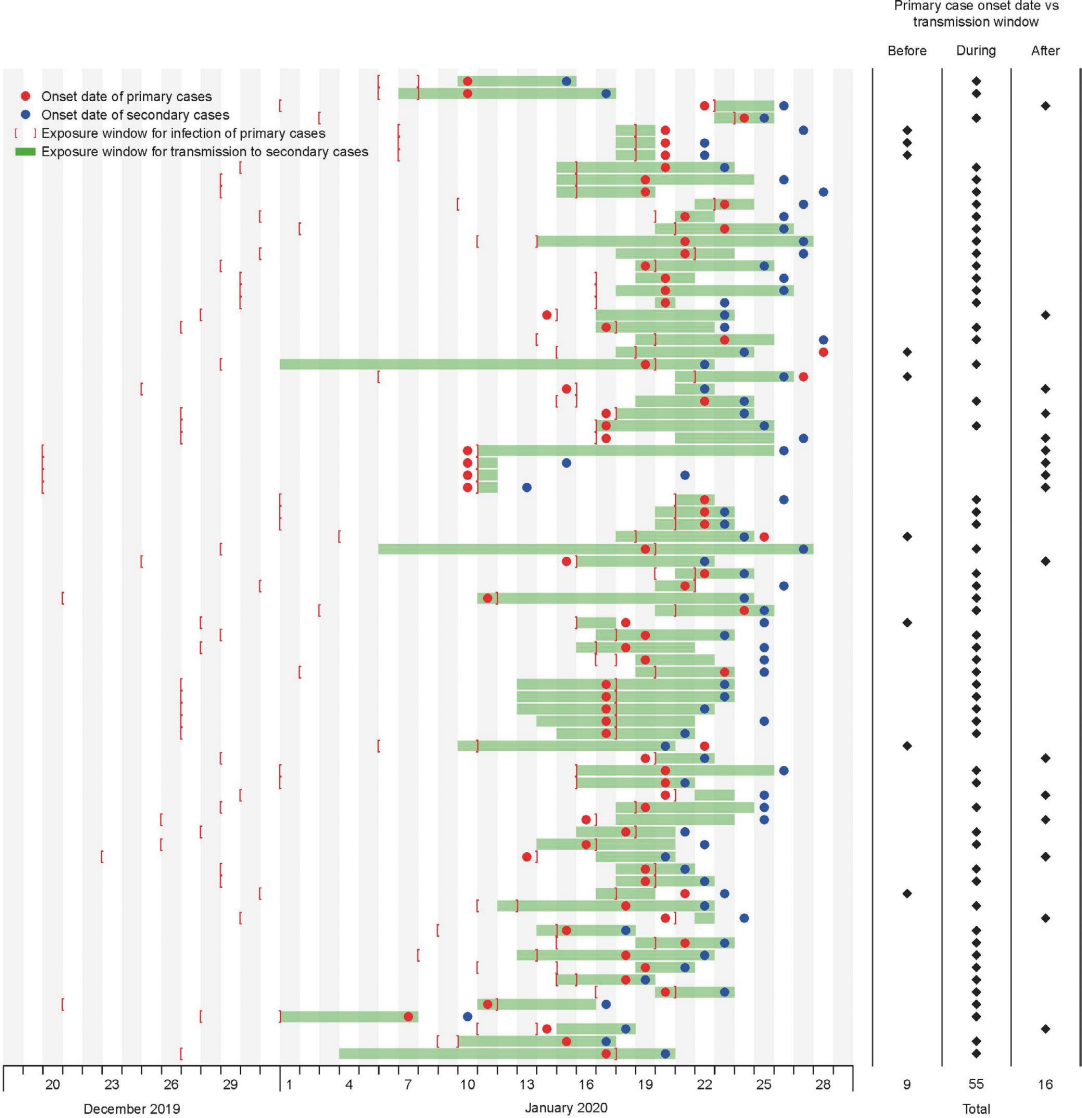
Transmission events used to infer the occurrence of pre‐symptomatic transmission. Dots indicate the dates of onset of primary and secondary cases, and the shaded area in each row indicates the period of exposure of the secondary case to the primary case during which the secondary infection is thought to have occurred. Brackets indicate the exposure window when the primary case was thought to have been infected. Data were resolved to the nearest day, and so transmission windows are plotted from the start of the first date to the end of the last date, onset dates are plotted in the middle of the corresponding day, and if the secondary onset date is the same as the primary onset date, then the former is offset slightly so that both can be seen

The identified 80 observations of transmission events were used for estimation of the serial interval (Figure 2B). We fitted a normal distribution to these data, with allowing 4 negative serial intervals and correcting for epidemic growth, and estimated that the serial interval distribution had mean 5.7 days (95% CI: 4.7, 6.8) and standard deviation 3.5 days (Figure 2B). In detailed investigations of contact patterns, we obtained information on the period when the secondary infection could have occurred, and related this to the illness onset date of the infector. Figure 3 shows that of 80 pairs identified, pre‐symptomatic transmission occurred in 9 pairs, post‐symptomatic transmission occurred in 16 pairs, and in the remaining 55 pairs, transmission could have occurred either before or after the corresponding infector's illness onset date. In these 55 pairs, we used Monte Carlo simulations based on the incubation period distribution described above to estimate that 23/55 of transmission events had greater than 50% chance to be pre‐symptomatic transmissions. Thus, in total we infer that 32/80 (40%) of the observed transmission events were likely or very likely to have occurred prior to the onset of symptoms in the infector.

We examined demographic and clinical characteristics of all the confirmed sporadic and clusters cases (Table 1). Age distributions were similar between the two groups of patients, while less male cases were identified in cluster secondary cases (43% vs 60%). Cluster primary and sporadic cases were generally more severe, with 26% and 7% in severe and critical conditions, respectively, compared with 12% and 1% in cluster secondary cases. Nearly all the primary and sporadic cases (96%) had a radiologic indication of pneumonia, compared with 74% in the secondary cases. Among 80 cluster secondary cases, 87% had been classified as mild cases, while 66% (110/166) of the cluster primary and sporadic cases were mild. The primary and sporadic cases showed largely similar onset symptoms as the secondary cases. Fever is the most common symptom, followed by headache, fatigue, dry cough and myalgia. Generally higher proportions of the cluster primary cases and sporadic cases reported an onset of the symptoms than cluster secondary cases (Table 1). In addition to systemic and respiratory presentations, a small proportion of the cases also reported gastrointestinal symptoms, including vomiting and diarrhoea.

**Table 1:** Characteristics of novel coronavirus infected patients identified in other provinces in China outside of Hubei through the Chinese Public Health Event Surveillance System as of 30 January 2020

Characteristics	Cluster primary cases and sporadic cases (n = 201)		Cluster secondary cases (n = 80)	
	Number	%	Number	%
Age, years				
<15	0/197	0	1/79	1
15‐44	96/197	49	37/79	47
45‐64	80/197	41	32/79	41
≥65	21/197	11	9/79	11
Male	120/200	60	34/80	43
Underlying conditions	29/95	31	6/18	33
Hypertension	12/95	13	4/18	22
Diabetes	6/95	6	1/18	6
Severity at day of analysis				
Mild	110/166	66	65/75	87
Severe	43/166	26	9/75	12
Critical	12/166	7	1/75	1
Death	1/166	0.6	0/75	0
Symptoms at illness onset				
Fever	168/183	92	57/64	89
Headache	26/29	90	6/10	60
Fatigue	70/96	73	20/31	65
Dry cough	75/109	69	25/38	66
Myalgia	43/73	59	14/26	54
Nausea	4/7	57	0/3	0
Chill	38/77	49	7/19	37
Sore throat	33/71	46	8/22	36
Chest distress	16/60	27	3/16	19
Dizziness	11/50	22	0/13	0
Tachypnoea	12/54	22	2/13	15
Loss of appetite	9/46	20	1/14	7
Dyspnoea	5/47	11	0/13	0
Diarrhoea	6/53	11	2/15	13
Vomiting	3/52	6	2/15	13
Pneumonia (radiology)	146/152	96	35/47	74
White blood cell count				
Decreased (<4 × 10^9^/L)	35/141	25	10/46	22
Normal (4‐10 × 10^9^/L)	102/141	72	35/46	76
Increased (>10 × 10^9^/L)	4/141	3	1/46	2
Lymphocyte count^a^				
Decreased	64/125	51	23/40	58
Normal	48/125	38	15/40	38
Increased	13/125	10	2/40	5

Note

For patients having clinical test results of both lymphocyte count and proportion, a normal lymphocyte count refers to both the lymphocyte count within the range of 1‐4 × 10^9^/L and the lymphocyte proportion to be 20%‐40%. Patients with either lymphoycyte count or lymphocyte proportion lower or higher than the normal range will be classified as “decreased” or “increased,” respectively.

^a^ Category of lymphocyte count is determined by both the count and proportion of lymphocyte (the proportion is derived as lymphocyte count divided by white blood cell count) in the blood test.

Based on the serial interval distribution with mean 5.7 days estimated above, we estimated that the effective reproductive number was 1.98 (95% CI: 1.68, 2.35). If instead we used a previous estimate of the serial interval distribution with mean 7.5 days and standard deviation 3.4 days, we would obtain an estimate of the effective reproductive number of 2.48 (95% CI: 1.90, 3.42).

## 4 DISCUSSION

In this study, we report estimates of the transmission dynamics of COVID‐19 based on cases identified outside of Hubei province. Importantly, we examine quantitative evidence for pre‐symptomatic infectiousness, a feature which complicates control strategies. We demonstrate that the phenomenon of pre‐symptomatic transmission is not uncommon, having occurred in a minimum of 9/80 transmission events (Figure 3). Using statistical inference based on our estimated incubation period distribution, we estimated that pre‐symptomatic transmission was likely to have occurred in up to 40% of the 80 transmission events in our data set. This observation should be interpreted in the context of isolation of some cases after illness onset, reducing the amount of post‐symptomatic transmission that might otherwise have occurred. This also implies that social distancing measures may be some of the most important strategies to reduce transmissibility, for example closing schools and encouraging, or facilitating working at home, in addition to control of onwards transmission by sequestering symptomatic persons at home or in isolation facilities.

Our finding was consistent with other reports that the serial interval distribution was similar to, or much lower than the incubation period distribution,^13^, ^18^, ^19^, ^20^, ^21^ consistent with the occurrence of pre‐symptomatic transmission in case reports.^22^, ^23^, ^24^, ^25^, ^26^

Pre‐symptomatic infectiousness is generally not thought to occur for most respiratory viruses, but measles is a well‐known example of a respiratory infection that can be spread before symptom onset,^27^ and viral shedding during the incubation period has also been reported for influenza.^28^ Viral shedding has been reported during the incubation period for COVID‐19,^29^ and there were also asymptomatic cases for COVID‐19 across all age groups, in varying proportions depending on the intensity of surveillance and testing.^30^, ^31^

In conducting investigations of clusters of cases, it is often found that secondary cases have on average milder illnesses than primary cases; for example, this was noted for Middle East Respiratory Syndrome coronavirus infections.^32^ Here, we found that secondary cases were more likely to have milder disease (Table 1), which would be consistent with the existence of some milder COVID‐19 infections, which were not laboratory confirmed. The cluster investigations that we report here are not likely to represent the full spectrum of mild infections, and better information would be provided by prospective studies of close contacts with repeated collection of respiratory swabs and sera.

Our estimates of the growth rate of infections in Wuhan in early January 2020 of around 0.14 per day (95% CI: 0.11, 0.17) are very consistent with previous reports.^4^, ^6^ Our estimate of the effective reproductive number in Wuhan is now slightly lower than the previous estimate of R_0_ ^4^ and some other estimates of R_0_ ^5^, ^6^, ^7^ but that is because of the shorter serial interval. We included a limited number of cases in other cities in China that were detected in persons that had not been to Wuhan (Figure 1B), but the sample size was insufficient at the time of analysis to determine whether there has been sustained transmission in any other cities.

The incubation period averaged 4.6 days and up to 11.1 days in 95% of infections (Figure 2A), which is important for specifying quarantine periods and also for understanding transmission dynamics. The city of Wuhan was put under lockdown on 23 January 2020, and other nearby cities were also locked down on the following days. This stopped exportation of infections to other Chinese cities, and the effect of this intervention has become more apparent in early February, given the delays that occur between infection, illness onset, admission to hospital and then laboratory testing.

We restricted the study participants to COVID‐19 cases reported outside Hubei Province in the early stage of outbreaks in China, before any community transmission had occurred in these areas. This selection of study time could allow us to identify infection sources as well as time of exposure to infections sources in a more robust way; otherwise, it would be much more difficult to identify human‐to‐human transmission chains based on epidemiological investigations.

Our study has a number of limitations. First, our findings were based on confirmed cases that will not capture every infection and would tend to be biased towards more severe infections. If the detection rate increased through January, for example because of greater availability of laboratory tests, we might have overestimated the growth rate and the effective reproductive number. Our estimate of the serial interval may be shorter if primary cases tended to isolate after illness onset, reducing the chance of onwards transmission with longer serial intervals, and our serial interval may also be slightly biased downwards because of exponential growth of the epidemic during this period. Similarly, we may have overestimated the role of pre‐symptomatic transmission if cases tended to take measures to reduce their infectivity after illness onset. Our data and analysis were also subject to ascertainment of symptom onset date, which was self‐reported by the patient and could be less reliable especially at the early stage of symptom presentation.

In conclusion, our analysis showed evidence indicative of pre‐symptomatic transmission of COVID‐19. Quarantine of exposed persons having close contact with infected cases before symptom onset could reduce the risk of further transmission.

## CONFLICT OF INTEREST

All the authors have declared no relationships or activities that could appear to have influenced this work. The researchers confirm their independence from funders and sponsors. The views expressed in this article are those of the authors and do not represent the official policy of the China CDC.

## AUTHOR CONTRIBUTIONS

**Xiang Ren:** Data curation (equal); Formal analysis (equal); Investigation (equal); Project administration (equal); Writing–original draft (equal); Writing–review & editing (equal). **Yu Li:** Conceptualization (equal); Data curation (equal); Formal analysis (equal); Investigation (equal); Methodology (equal); Project administration (equal); Writing–original draft (equal); Writing–review & editing (equal). **Xiaokun Yang:** Data curation (equal). **Zhili Li:** Data curation (equal). **Jinzhao Cui:** Data curation (equal). **Aiqin Zhu:** Data curation (equal). **Hongting Zhao:** Data curation (equal). **Jianxing Yu:** Formal analysis (equal). **Taorao Nie:** Data curation (equal). **Minrui Ren:** Data curation (equal). **Shuaibing Dong:** Data curation (equal). **Ying Cheng:** Writing–review & editing (equal). **Qiulan Chen:** Writing–review & editing (equal). **Zhaorui Chang:** Writing–review & editing (equal). **Junling Sun:** Writing–review & editing (equal). **Liping Wang:** Writing–review & editing (equal). **Luzhao Feng:** Writing–review & editing (equal). **George Fu Gao:** Writing–review & editing (equal). **Zijian Feng:** Writing–review & editing (equal). **Zhongjie Li:** Conceptualization (equal); Funding acquisition (equal); Resources (equal); Supervision (equal); Writing–review & editing (equal).
